# Application of the PDCA cycle for standardized nursing management in sepsis bundles

**DOI:** 10.1186/s12871-022-01570-3

**Published:** 2022-02-04

**Authors:** Chunxia Liu, Yun Liu, Yiqing Tian, Kun Zhang, Guizhen Hao, Limin Shen, Quansheng Du

**Affiliations:** 1 Department of ICU, Hebei General Hospital, No. 348 Heping West Road, Shijiazhuang, 050051 China; 2 Department of Quality control office, Hebei General Hospital, Shijiazhuang, 050051 China

**Keywords:** PDCA, Sepsis, Bundle therapy, Compliance

## Abstract

**Background:**

To explore the application effect of plan, do, check and action circulation management mode in improving the compliance of sepsis bundle treatment.

**Methods:**

113 patients with sepsis admitted from January 1 to December 31, 2018 were selected as the control group, and the bundle treatment measures of sepsis were routinely implemented. The above treatment measures were completed within 6 h. 113 patients with sepsis admitted from January 1 to December 31, 2019 were selected as the study group. All clinical staff took the same measures as the control group, supplemented by PDCA cycle management. Objective to compare the changes of compliance of clinical staff to sepsis bundle treatment before and after the implementation of PDCA cycle management.

**Results:**

Compared with the control group, the study group achieved the completion rate of sepsis bundle treatment in 1 h from 66.4 to 81.4%, the completion rate in 3 h from 77.0 to 89.4%, and the completion rate in 6 h from 82.3 to 95.6%. The difference was statistically significant (*P* < 0.05 for all).

**Conclusions:**

The implementation of PDCA cycle management mode can effectively improve the compliance of clinical staff to the bundle treatment of sepsis, improve the treatment efficiency of sepsis, and improve the quality of medical care.

**Supplementary Information:**

The online version contains supplementary material available at 10.1186/s12871-022-01570-3.

## Background

The *Chinese Guidelines for the Emergency Treatment of Sepsis/Septic Shock (2018)* defines sepsis as life-threatening organ dysfunction caused by a dysregulated host response to infection [1]. Prompt diagnosis and treatment of sepsis is very important, and according to the World Health Organization, sepsis should be treated as a priority by global health systems [2]. More than 80% of patients survive when shock is treated within 1 h; if shock is diagnosed and treated after 6 h, the survival rate drops to 30% [3, 4]. The Surviving Sepsis Campaign (SSC) is a joint initiative by the European Society of Intensive Care Medicine and the Society of Critical Care Medicine that is dedicated to reducing the morbidity and mortality of sepsis and septic shock worldwide. Sepsis bundles have always been the core strategy of the SSC guidelines. It emphasizes the necessity and importance of timely and effective implementation of cluster therapy within 1 h for septic shock. It has been proven by many countries that it can significantly improve the prognosis of patients with sepsis and septic shock and has been considered a cornerstone for improving the quality of treatment of sepsis and septic shock since 2005 [5–7]. However, there remains a gap between guideline recommendations and clinical practice. The overall compliance during the course of cluster therapy is low and there is a large variation in the attainment rate among the items of the treatment bundles recommended by the guidelines with a completion rate of 23.5% (16/68) for cluster therapy within 3 h after septic shock and 33.3% (20/68) within 6 h [8]. Poor adherence to guidelines and poor implementation by staff directly increase the 28d mortality of patients with severe sepsis and septic shock [9], which requires multidisciplinary awareness and compliance. At present, the methods to improve the compliance of sepsis bundles include the establishment of departmental medical and nursing teams for sepsis treatment, the use of checklists, training, assessment and educational supervision, but the highest reported rate of adherence to the standard in China is only about 81% [10, 11]. The Plan, Do, Check, Act (PDCA) Cycle, also known as the Deming Cycle, can assist clinical staff in clinical work to proactively identify problems, strictly link quality control and management and optimize workflow [12, 13]. The PDCA method gradually improves the quality of work through a closed loop system and circular management of improvement projects in four stages: Plan, Do, Check, and Act [14]. The clinical use of PDCA for management can not only ensure more rigorous and effective medical and nursing practices, but also improve medical and nursing quality [15]. PDCA cycle management is effective in improving the compliance of clinical staff to sepsis bundles.

## Methods

### General data

#### Study subjects

113 septic patients admitted to the Department of Critical Care Medicine of our hospital from January 1 to December 31, 2018 were selected as the control group; 113 septic patients admitted from January 1 to December 31, 2019 were selected as the study group.

#### Inclusion criteria

Meeting the diagnostic criteria of the *Chinese Guidelines for the Treatment of Severe Sepsis/Septic Shock (2014)* by the Chinese Society of Intensive Care Medicine Branch [3], and admission to the ICU to confirm the diagnosis of sepsis with a duration of stay longer than 6 h. Patients were diagnosed with severe sepsis upon arrival or in the ICU unit. Severe sepsis was diagnosed by the physician and usually took less than 1 h from the time the patient has symptoms to the time of diagnosis. Severe sepsis was diagnosed as sepsis with organ dysfunction and/or inadequate tissue perfusion (any of the following): 1) hypotension due to sepsis; 2) lactate level exceeding the upper limit of the normal level of laboratory tests; 3) urine volume < 0.5 mL/(kg·h) for at least 12 h even with adequate fluid resuscitation; 4) acute lung injury due to non-pneumonia and oxygenation index < 250 mmHg; 5) acute lung injury due to pneumonia and oxygenation index < 200 mmHg; 6) blood creatinine > 176.8 μmol/L; 7) serum bilirubin > 34.2 μmol/L; 8) platelet count < 100 × 10^9^/L; or 9) coagulation disorder (international standardized ratio > 1.5). The diagnostic criteria for septic shock were sepsis with sepsis-induced hypotension which cannot be reversed by fluid therapy.

#### Exclusion criteria

Termination of treatment, discharge or death within 6 h after admission.

#### Ethics

The study was approved by the hospital’s medical ethics committee; approval number (2020), ethical review No. 108. The Informed Consent Form was signed with the patient’s family and the subject could voluntarily terminate their participation in the study at any time and would not be prevented from receiving further treatment.

### Study methods

A prospective cohort study was used.

#### Control group

We tried to use the bundle therapy in all patients, but it cannot be achieved in practice. The optimal approach (bundle) in sepsis treatment in the control group is described as follows. As required by the *Chinese Guidelines for the Treatment of Severe Sepsis/Septic Shock* and the *Professional Quality Control Index for Critical Care Medicine (2015)*, the physician diagnosed sepsis and notified the nurse. The nurse immediately measured temperature, blood pressure, central venous pressure (CVP), and central venous oxygen saturation (ScvO_2_) and inserted an indwelling urinary catheter. The doctor gave medical orders for antimicrobial infusion, rehydration, application of antihypertensive drugs, collection of blood culture, blood gas analysis, blood routine, calcitonin, and other specimens. The nurse immediately carried out these orders and closely observed the improvement of blood pressure, urine volume, skin endings, etc. The doctors and nurses reminded each other to complete the above treatment measures within 6 h. On the basis of bundle, we introduced the PDCA cycle into the study group.

#### The sepsis treatment team for study

Group A was established in the department and all clinical staff under the leadership of medical and nursing team leaders applied PDCA cycle management to the problems in the sepsis bundles. Specific measures were as follows:

##### Plan (P)

**Fig. 1:**
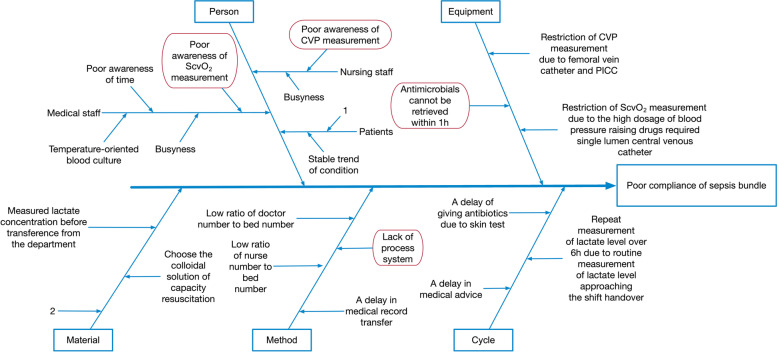
The fishbone diagram of cause analysis. 1: Financial trouble and difficulties to make use of PICCO and so on for target capacity resuscitation. 2: Supplied crystalloid solution before transference from the department

113 cases of sepsis from January to December 2018 were retrospectively investigated. The problems identified in the process of cluster therapy were that antibiotics could not be given in time, the retention rate of blood culture before the application of antibiotics was low, the measurement of ScvO_2_ and CVP was delayed or not measured, the measurement of lactic acid was delayed, and the volume of fluid and dosage did not meet the guideline requirements. The main reasons for poor adherence were analyzed as busy clinical staff, insufficient knowledge of guidelines, poor awareness of ScvO_2_ and CVP measurement, lack of antimicrobial stockpiles in the department, and a delay in medical record transfer and order creation resulting in a medication time greater than 1 h. The causes of poor adherence were plotted into a fishbone diagram of cause analysis (see Fig. 1). Group members used evaluation methods and other means to identify the main causes and highlighted them with red circles on the fishbone diagram.

##### Development (D)

The improvement strategies were completed after January 2019 while their development process was earlier than 2019. Corresponding improvement strategies were introduced for different causes and clinical staff continued to implement the cluster therapy strategies for sepsis on the basis of improvement. Specific improvement measures are shown in Supplementary Table S1. There were several examples: we enhanced doctors and nurses’ compliance of implementing sepsis bundle therapy by training them on the importance and necessity of sepsis bundle therapy. Nursing team leaders, matrons and medical team leaders were responsible for supervision and quality control. Reward and punishment scheme: at the beginning of each month, statistics on the completion of sepsis bundle therapy in the previous month were compiled, and criticism is made at the morning meeting and departmental quality control meeting for those who do not complete well, and performance penalties are given to those who fail to implement measures due to subjective reasons such as forgetfulness; praise and rewards were given to those who complete well. Information technology, such as the critical care intelligent clinical decision system, can be used to help improve compliance with sepsis bundle therapy.

##### Check (C)

After the implementation of improvement measures, the completion rates of the sepsis bundles for 1 h, 3 h, and 6 h were calculated, respectively.

##### Assessment (A)

Standardize the process of sepsis cluster treatment (see Supplementary Table S2) and continuously evaluate future implementation processes to ensure compliance with the indications, the interventions and the effectiveness of the measures. The compliance reflected the willingness of the medical staff to use bundle therapy while the completion rate reflected the final completion of the bundle therapy.

### An evaluation index

An evaluation index was used to compare the completion rates for 1 h, 3 h, and 6 h for sepsis bundles in the study and control groups.

### Statistical methods

The data used in this study were analyzed using SPSS 18.0. The comparison of the count data was performed by the χ2 test and the mean ± standard deviation (x±s) was used for statistical description. The t-test and ANOVA (with necessary correction in case of variance) were used for the comparison of differences between groups, F-values were calculated, and a *P*-value under 0.05 was considered statistically significant.

## Results

### General information

There was no statistically significant difference between the two groups in terms of age, gender, APACHE II score, and primary diseases; *P* > 0.05 for all (see Table 1).

**Table 1:** Comparison of general information of patients in two groups

Groups	Cases	Gender (n)		Age (years)	APACH II score	Primary diseases, n (%)				
	(n)	Male	Female			Abdominal infection	Pulmonary infection	Hematologic infection	Urinary tract infection	Other infection
Control group	113	62	51	77.24 ± 6.91	20.13 ± 5.18	41 (36)	28 (25)	19 (17)	12 (11)	13 (11)
Study group	113	63	50	78.73 ± 7.22	21.48 ± 6.35	39 (35)	32 (28)	17 (15)	14 (12)	11 (10)
χ^2^ /t		0.018		1.733	1.918	0.748				
P		0.894		0.084	0.056	0.945				

*APACH* Acute Physiology and Chronic Health Evaluation

### Comparison of the compliance of the two groups of patients with septic shock bundle treatment measures

Improved indicators within 1 h were blood culture (from 72.6 to 84.1%, *P* = 0.036) before antibiotic treatment, administration of broad-spectrum antibiotics (from 73.5 to 85.0%, *P* = 0.033), and ScvO_2_ (from 66.4 to 81.4%, *P* = 0.010) measurement; Improved indicators within 3 h were blood culture (from 69.9 to 82.3%, *P* = 0.029) before antibiotic treatment, administration of broad-spectrum antibiotics (from 91.1 to 98.2%, *P* = 0.018), and ScvO_2_ (from 77.0 to 89.4%, *P* = 0.013) measurement; Improved indicator within 6 h was ScvO_2_ (from 82.3 to 95.6%, *P* = 0.001) measurement. With the passage of time, the compliance of blood culture before antibiotic treatment, administration of broad-spectrum antibiotics, administration of 30 ml/kg crystalloid solution for target resuscitation when hypotension or lactic acid ≥4 mmol/L, CVP measurement, ScvO_2_ measurement and repeated lactic acid measurement were improved to varying degrees. Doctors and nurses were more aware of blood gas checking, and measuring lactic acid concentration is easy to perform. When patients had low blood pressure, the first response of doctors and nurses was to give vasopressor drugs. Therefore, compliance with these two indicators was relatively good from beginning to end (see Supplementary Table S3).

### Completion rates of 1 h, 3 h and 6 h of sepsis bundles in both groups (see Table 2)

After the implementation of PDCA cycle management in the study group, the completion rate of the one-hour sepsis bundles increased from 66.4 to 81.4%, the three-hour completion rate increased from 77.0 to 89.4%, and the six-hour completion rate increased from 82.3 to 95.6%. These produced a *P*-value < 0.05, meaning the differences were statistically significant. The compliance of the sepsis bundles had significantly improved in the study group compared with the control group.

**Table 2:** Comparison of 1 h, 3 h, 6 h completion rates of septic shock bundle treatment (bundle) between the two groups of patients (%)

Group	Number of cases	1 h completion rate(%)	3 h completion rate(%)	6 h completion rate(%)
Control group	113	75 (66.4)	87 (77.0)	93 (82.3)
Study group	113	92 (81.4)	101 (89.4)	108 (95.6)
χ^2^		6.629	6.200	10.119
P		0.010	0.013	0.001

### Comparison of the prognosis of the two groups

The effect indicators of 6 h bundle treatment for the two groups of patients: MAP, CVP, ScvO_2_, 6 h lactic acid clearance rate (LCR), urine output, norepinephrine dose, etc. Outcome indicators of the two groups of patients: ICU hospital stay and 28-day mortality rate.

#### Results indicators of 6 h septic shock bundle treatment (Table 3)

There was no statistically significant difference between study and control groups in MAP, CVP, or urine output. Compared with the control group, the study group had statistically higher ScvO_2_ (72.56 ± 4.23 vs.70.68 ± 5.15) and 6 h lactate clearance rate (37.35 ± 6.98 vs. 34.23 ± 7.23), and a lower norepinephrine dose (0.79 ± 0.37 vs. 0.93 ± 0.25), *P* < 0.05 for all.

**Table 3:** Comparison of the effect indicators of the two groups of patients after 6 h of bundle treatment

Indicator	Study group(*n* = 113)	Control group (*n* = 113)	t	*P*
MAP (mmHg)	74.15 ± 8.98	72.33 ± 9.97	1.442	0.151
CVP (mmHg)	9.98 ± 3.37	10.25 ± 3.85	0.561	0.575
ScvO_2_(%)	72.56 ± 4.23	70.68 ± 5.15	2.999	0.003
6 h LCR(%)	37.35 ± 6.98	34.23 ± 7.23	3.300	0.001
Urine output (mL/h)	41.38 ± 13.13	43.56 ± 12.56	1.275	0.203
Norepinephrine dose (μg·kg^−1^·min^−1^)	0.79 ± 0.37	0.93 ± 0.25	3.333	0.001

6 h lactic acid clearance rate LCR (%) = [Lac(T0)-Lac(T6)]/Lac(T0) × 100%

*CVP* central venous pressure, *LCR* lactic acid clearance rate, *ScvO*_2_ Central venous oxygen saturation

#### Outcome indicators of septic shock bundle treatment (Table 4)

Compared with the control group, the study group had shorter ICU hospital stay (7.97 ± 2.76 vs. 9.25 ± 2.83) and the difference was statistically significant (*P* = 0.001). There was no significant difference in 28-day mortality rate between the two groups of patients (*P* = 0.091).

**Table 4:** Comparison of outcome indicators of bundle treatment between the two groups of patients

Group	Number of case(n)	ICU hospitalization length (d, $\overline{\chi}$ ±s)	28-day mortality rate %(cases)
Control group	113	9.25 ± 2.83	25.7 (29)
Study group	113	7.97 ± 2.76	16.8 (19)
χ^2^ /t		3.442	2.858
*P*		0.001	0.091

### Clinical practice

Naturally, three concise bullet points of the significance of clinical nursing can be concluded. The first point is, with the application of PDCA, the importance and necessity of sepsis bundles therapy can be more profound understood by nursing and medical staff, and they will pay more attention to sepsis bundles therapy. The key thing is to acknowledge the multi-disciplinary and time-dependent nature of sepsis management, and knowledge of the bundles allows clinicians, nursing, and medical staff to work more effectively together. After that the sepsis bundles patients are admitted to the hospital, nurses are consciously able to work according to the process of sepsis bundles therapy. Meanwhile, the phenomenon of waiting for medical advice is overturned, which ensures that all the programs of sepsis bundles therapy are put into practice one by one and ensures to improve the attainment rate of sepsis bundles therapy. The second point is that it provides a system guarantee and basis for the timely and effective practice of sepsis bundles therapy that establishment and execution of management system of sepsis bundles therapy and the foundation of the department sepsis bundles treatment group including doctors and nursing staff in which the medical personnel who are positive, professional and interested in the treatment and care for sepsis bundles take part. Each shift has at least 1 member of the department sepsis bundles treatment group, which improves the other medical personnel’s positiveness and the compliance of carrying out the system and process in the department. In addition, the compliance and implementation rate of the management system of sepsis bundles therapy is further improved under the supervision of the head nurse towards the executive condition of the management system of sepsis bundles. The third point is, with the application of specialized supervision, quality control, and the reward and punishment plan, it is improved that the medical personnel’s sense of responsibility, positiveness and initiative, and enthusiasm of making use of sepsis bundles therapy despite any difficulty.

### Implications for clinical practice

According to the PDCA cycle management model, clinical nursing practice has been changed. Firstly, once sepsis disease was diagnosed by doctors, the nursing staff would list the implementation schedule, reminding themselves when and which measures should be implemented, and ask colleagues or group leaders for help on the same shift if they couldn’t solute it by themselves. Secondly, they will try their best to squeeze more time and energy to concentrate on timely and effective practice of all the programs of bundles therapy while doing well in daily care. Thirdly, the responsible nurse would remind busy doctors who may forget something need to do what should be done next, which overturns the phenomenon of waiting for medical advice. Finally, more attention was paid to projects with poor completion rates.

To improve the effective and timely implementation of sepsis bundle treatment in the Department of Critical Care Medicine, a management system is formulated for improving the compliance. The details were as follows: 1) the department established a medical and nursing rescue team for sepsis bundle treatment, with one medical team leader, one nursing team leader and 18 team members, including 4 doctors and 14 nurses; 2) The team members were distributed to each responsible nursing group and relatively fixed to ensure that there was one nursing member of the treatment team on duty in each shift in each ward; 3) The members of the rescue team were trained at least once a month on knowledge related to sepsis bundle treatment; 4) The rescue team hold a quality control analysis meeting once a month and invited the department chief and nurse manager to participate, so as to summarize and analyze the key and difficult problems in the process of sepsis rescue and treatment and put forward improvement opinions; 5) The medical team leader and nursing team leader of the sepsis treatment team together with the head nurse regularly conduct quality control supervision and inspection on the compliance of sepsis bundle treatment; 6) A corresponding reward and punishment system was developed to give different degrees of rewards and punishments to doctors and nurses who complete better and those who do not complete as required every month according to the objective situation after discussion and deliberation of the assessment team. If the measures not implemented or not implemented due to subjective forgetfulness, one point will be given as performance penalty; for better completion of sepsis centralized treatment compliance, one point will be given as performance reward for the highest completion rate in that month.

## Discussion

Since 2004, international sepsis guidelines have been updated four times, domestic guidelines have been launched successively, quality control standards for sepsis diagnosis and treatment have been improved and the optimal time period for cluster therapy has been adjusted from 3 h and 6 h to 1 h. This was proposed in 2018, which has put forward higher requirements for standardized diagnosis and treatment of sepsis, comprehensive management of critically ill patients by medical institutions, and coordination among hospital departments [16, 17]. The completion rate of sepsis bundles has become one of the criteria for quality control of critical care by hospital management [18]. The New York Centers for Medicare and Medicaid Services in the United States require hospitals to report sepsis cluster therapy performance rates to them as part of the inpatient quality reporting program and as a condition of payment [19]. Therefore, it is necessary to correctly calculate the completion rates of sepsis cluster therapy at 1 h, 3 h, and 6 h, and to take effective measures to continuously improve the completion rates. Despite the various measures taken to improve compliance for sepsis cluster therapy, the attainment rate is still unsatisfactory [10, 11]. In this study, the PDCA cycle management model was adopted, in which the medical and nursing team leaders regularly informed and analyzed data on the compliance of sepsis bundles, summarized the problems and difficulties in the implementation process and the sepsis treatment team members then discussed and formulated corresponding countermeasures. According to the inspection, additional points were rewarded or deducted on the basis of the original performance. This cycle is repeated, which promotes the effective operation of PDCA cycle management, improves the sense of responsibility and urgency of clinical staff and ensures the improvement of sepsis bundle compliance. The completion rates of 1 h, 3 h and 6 h for sepsis cluster treatment reached 81.4, 89.4, and 95.6%, respectively, which is related to the fact that the department has repeatedly trained staff and emphasized the importance of cluster treatment for 3 years.

Additionally, the compliance of treatment has improved to a certain extent through methods such as checklists. However, due to the existence of objective reasons, such as low bed-to-nurse ratios and delayed transfer of medical records, further improvement of clinical staff compliance to sepsis cluster therapy needs to be addressed in terms of rationalization and maximization of ICU human resource allocation, optimization of the referral process and medication pick-up process. One-hour cluster therapy can achieve the goal of reducing 28d morbidity and mortality rate [20], but mandatory rapid use of broad-spectrum antimicrobials, especially in patients without shock, may lead to their overuse [21], a viewpoint that influences some physicians’ prescription of broad-spectrum antimicrobials and contributes to the low overall treatment adherence rate.

Tissue hypoperfusion is an important factor that aggravates the condition and leads to death in patients with septic shock. Both LCR and ScvO_2_, which reflect tissue perfusion, can be used to evaluate the condition of patients with septic shock and to judge the effect of early resuscitation treatment [22]. Applied sepsis shock cluster ScvO_2_ patients after treatment of clinical nursing path and the LCR tissue perfusion index improved significantly, vascular active drug norepinephrine reduced dosage, ICU hospitalization time shortened, but there was no significant difference, the fatality rate of 28 days that sepsis shock cluster the treatment of clinical nursing path can improve the treatment of patients with sepsis shock effect, can improve the prognosis of patients with the ending [22].

We listed all the medical care contents involved and required within 6 h from the patient’s admission to the ICU to confirm the diagnosis of septic shock, used the form to tick, and designed a clinical care pathway for sepsis, which was guided by evidence-based care and emphasized the standardization of care for septic shock bundle treatment under the guidance of guidelines, and can promote the effective implementation of septic shock bundle treatment [23]. The Critical Care Intelligent Clinical Decision System helped medical staff to identify and diagnose septic shock at an early stage by establishing a sepsis early warning and treatment mechanism, and by compiling and analyzing the data obtained, making clinical decision early warning and using red words to remind doctors to make diagnosis in time. The systematized process was embedded into the quality control module of the critical care information system. For each stage, medical staff was reminded in a timely and repeated manner according to the set process and content, so as to urge them to complete treatment measures within a specific period of time, which can avoid their negligence and forgetfulness due to busy schedule and weak sense of responsibility, overcome their subjective factors of poor compliance with septic shock bundle treatment, and ensure the implementation of septic shock bundle treatment measures at 1 h, 3 h and 6 h, improving the treatment effect of patients with septic shock [24]. A checklist was used to design the bundle treatment plan into a form, with each item filled in by ticking or forking. Compared with the conventional measures, it can remind health care workers to timely and accurately implement relevant regulations and policies during the busy process of treating critically ill patients, with clear target and purpose, which can effectively improve the compliance of health care workers to standardize the implementation of operations, and facilitate managers to supervise the effect. Overall, it is an effective way to improve the success rate of infectious shock treatment [25].

There are several limitations in our study. First, this study may be inherently influenced by potential selection bias due to its retrospective design. To some extent, objective reasons hindering the implementation of cluster therapy and subjective factors exist, such as cognitive bias and poor practice of cluster therapy by medical personnel, which makes 100% compliance of sepsis cluster therapy difficult to reach. The sample size of this study is relatively small and regional in nature, and as an observational cohort study it also has its inherent limitations and biases. We will continue to conduct in-depth multidisciplinary and multicenter studies on adherence to the processes for sepsis bundles to provide more bases for clinical decisions.

## Conclusions

The PDCA cycle management model, in which existing and potential problems are identified in clinical work, a problem-based improvement plan is developed, corresponding measures are implemented strictly according to the rectification plan and the results of implementation and execution are checked, standardized, or process-oriented, and the above links are cycled back and forth to better highlight the advantages of continuous improvement in quality management, continuously improve the quality of medical care and ensure medical safety [26].

## Supplementary Information

**Additional file 1: Table S1.** Countermeasures for poor compliance of sepsis bundle.

**Additional file 2: Table S2.** Flow of sepsis bundle.

**Additional file 3: Table S3.** Comparison of compliance with treatment indicators of septic shock bundle treatment between the two groups (*n* = 226).

## Data Availability

All data generated or analyzed during this study are included in this published article.

## Abbreviations

PDCA: The Plan, Do, Check, Action
CVP: Central venous pressure
ScvO_2_: Central venous oxygen saturation
SSC: Saving Sepsis Campaign
