# Supplementary material for: Application of the PDCA cycle for standardized nursing management in sepsis bundles
Source: BMC Anesthesiol. 2022 Feb 4;22:39. doi: 10.1186/s12871-022-01570-3 (PMC8815114; doi:10.1186/s12871-022-01570-3)
Supplement: Supplementary file 1 — Additional file 1: Table S1. Countermeasures for poor compliance of sepsis bundle. [file 12871_2022_1570_MOESM1_ESM.docx]

**Table S1. Countermeasures for poor compliance of sepsis bundle**

| Problems | Counter measures |
| --- | --- |
| Poor awareness of doctors in ScvO2 measurement | 1. Strengthen the awareness of doctors and nurses on the importance and necessity of sepsis centralized treatment.   2. Formulate the process of sepsis centralized treatment and emphasize the joint compliance of doctors and nurses.  3. Dedicated personnel for supervision and quality control.  4. Develop a reward and punishment program. |
| Poor awareness of nurses in active CVP measurement |  |
| Antimicrobials cannot be retrieved within 1h | 1. Apply to the pharmacy department to reserve commonly used antimicrobials as base drugs.  2. Communicate with the pharmacy department to be able to temporarily borrow medication when the department's reserve is insufficient or cannot meet treatment needs, and then return the borrowed medication after the medical records are transferred and the medical orders are promptly made up.  3. When the preparation is insufficient, the nursing team leader is responsible for having the antimicrobial agent ready at the first time after the diagnosis of sepsis is confirmed.  4. For antimicrobials requiring skin tests, the deputy team leader on duty is responsible for the configuration of the skin test solution, implementation of the skin test and observation of the results. |
| Lack of system process | 1. Develop a departmental management system for improving compliance with sepsis bundle.  2. Formulate a flow chart of sepsis bundle.  3. Dedicated person is responsible for supervision and quality control of the implementation of the established system and process.  4. Set up a sepsis treatment team including doctors and nurses, and have team members on duty in every shift to ensure the timeliness and effectiveness of sepsis bundle. |

CVP: central venous pressure; ScvO2: central venous oxygen saturation.
