# Supplementary material for: Application of the PDCA cycle for standardized nursing management in sepsis bundles
Source: BMC Anesthesiol. 2022 Feb 4;22:39. doi: 10.1186/s12871-022-01570-3 (PMC8815114; doi:10.1186/s12871-022-01570-3)
Supplement: Supplementary file 2 — Additional file 2: Table S2. Flow of sepsis bundle. [file 12871_2022_1570_MOESM2_ESM.docx]

**Table S2. Flow of sepsis bundle**

| Time | Admission to ICU 1h | Admission to ICU 3h | Admission to ICU 6h |
| --- | --- | --- | --- |
| Indicators to be completed | □ Assess the condition and execute emergency medical orders, such as cooperate with doctors for tracheal intubation  □ Establish dual fluid lines with ≤22G indwelling needle, and establish central venous access with the physician  □ perform invasive blood pressure monitoring by puncturing artery  □ collect various specimens, especially blood gas, and measure blood lactate concentration  □ Perform blood culture before applying antimicrobial drugs  □ give broad-spectrum antibacterial drug treatment  □ Resuscitate with 30ml/kg crystalloid solution as prescribed for hypotension or lactate ≥4mmol/L | □ Continue the items not completed within 1h, especially  ○Measure blood lactate concentration  ○Blood culture before applying antimicrobial drug treatment  ○Treat with broad-spectrum antibacterial drugs  ○Give 30ml/kg crystalloid for target resuscitation for hypotension or lactate ≥4mmol/L  □ Perform other monitoring, treatment and care | □Continue the program within 3h  □Give blood pressure raising drugs  □Measure CVP  □ Measure ScvO2  □ Repeat measurement of blood lactate level |

CVP: central venous pressure; ScvO2: central venous oxygen saturation.
