# Supplementary material for: Application of the PDCA cycle for standardized nursing management in sepsis bundles
Source: BMC Anesthesiol. 2022 Feb 4;22:39. doi: 10.1186/s12871-022-01570-3 (PMC8815114; doi:10.1186/s12871-022-01570-3)
Supplement: Supplementary file 3 — Additional file 3: Table S3. Comparison of compliance with treatment indicators of septic shock bundle treatment between the two groups (n = 226). [file 12871_2022_1570_MOESM3_ESM.docx]

**Table S3 Comparison of compliance with treatment indicators of septic shock bundle treatment between the two groups (n=226)**

| Indicator | | Lactic acid concentration measurement | Blood culture before antibiotic treatment | Administration of broad-spectrum antibiotics | Administration of 30ml/kg crystalloid solution for target resuscitation when hypotension or lactic acid ≥4mmol/L | Administration of vasopressor drugs | CVP measurement | ScvO2 measurement | Repeated lactic acid measurement |
| --- | --- | --- | --- | --- | --- | --- | --- | --- | --- |
| 1hcompliance | Control group n=113 | 111(98.2) | 82(72.6) | 83(73.5) | 80(70.8) | 111(98.2) | 90(79.6) | 75(66.4) | 89(78.8) |
|  | Study group n=113 | 112(99.1) | 95(84.1) | 96(85.0) | 86(76.1) | 113(100) | 95(84.1) | 92(81.4) | 96(85.0) |
|  | χ^2^ | 0.338 | 4.404 | 4.540 | 0.817 | 2.018 | 0.745 | 6.629 | 1.460 |
|  | P | 0.561 | 0.036 | 0.033 | 0.366 | 0.155 | 0.388 | 0.010 | 0.227 |
| 3hcompliance | Control group n=113 | 113(100) | 79(69.9) | 103(91.1) | 82(72.6) | 113(100) | 102(90.3) | 87(77.0) | 99(87.6) |
|  | Study group n=113 | 113(100) | 93 (82.3) | 111(98.2) | 93(82.3) | 113(100) | 101(89.4) | 101(89.4) | 105(92.9) |
|  | χ2 | 0.000 | 4.769 | 5.632 | 3.064 | 0.000 | 0.048 | 6.200 | 1.813 |
|  | P | 1.000 | 0.029 | 0.018 | 0.080 | 1.000 | 0.826 | 0.013 | 0.178 |
| 6hcompliance | Control group n=113 | 113(100) | 88(77.9) | 113(100) | 100(88.5) | 113(100) | 108(95.6) | 93(82.3) | 107(94.7) |
|  | Study group n=113 | 113(100) | 99(87.6) | 113(100) | 106(93.8) | 113(100) | 110(97.3) | 108(95.6) | 112(99.1) |
|  | χ2 | 0.000 | 3.750 | 0.000 | 1.975 | 0.000 | 0.518 | 10.119 | 3.686 |
|  | P | 1.000 | 0.053 | 1.000 | 0.160 | 1.000 | 0.472 | 0.001 | 0.055 |

CVP: central venous pressure; ScvO2: central venous oxygen saturation.
